# School absenteeism in children with special health care needs. Results from the prospective cohort study ikidS

**DOI:** 10.1371/journal.pone.0287408

**Published:** 2023-06-23

**Authors:** Jennifer Schlecht, Jochem König, Stefan Kuhle, Michael S. Urschitz

**Affiliations:** 1 Division of Pediatric Epidemiology, Institute of Medical Biostatistics, Epidemiology and Informatics, University Medical Center Mainz, Mainz, Germany; 2 Perinatal Epidemiology Research Unit, Departments of Obstetrics & Gynaecology and Pediatrics, Dalhousie University, Halifax, Nova Scotia, Canada; Kyung Hee University School of Medicine, REPUBLIC OF KOREA

## Abstract

**Objective:**

Children with special health care needs (SHCN) due to a chronic health condition perform more poorly at school compared to their classmates. There is still little knowledge on the causal pathways and which factors could be targeted by interventions. We, therefore, investigated school absenteeism in children with SHCN compared to their peers.

**Methods:**

This study was based on data from the German population-based prospective cohort study ikidS (German for: I will start school). Children with SHCN were identified by the Children with Special Health Care Needs screener that captures five consequences of physical or mental chronic health conditions: (1) use or need of prescription medication, (2) above average use or need of medical, mental health, or educational services, (3) functional limitations compared with others of the same age, (4) use or need of specialized therapies, and (5) treatment or counseling for emotional, behavioral, or developmental problems. School absenteeism was defined as days absent from school due to illness during first grade and was reported by classroom teachers. Associations between SHCN consequences and school absenteeism were investigated by negative binomial regression models. Effect estimates were adjusted for confounding variables identified by a causal framework and directed acyclic graphs.

**Results:**

1,921 children (mean age at follow-up 7.3 years, standard deviation 0.3; 49% females) were included; of these, 14% had SHCN. Compared to their classmates, children with SHCN had more days absent (adjusted rate ratio: 1.37; 95% confidence interval 1.16, 1.62). The effect was strongest among children with i) functional limitations, ii) treatment or counseling for emotional, behavioral, or developmental problems, and iii) those who experienced two or more SHCN consequences.

**Conclusions:**

Children with SHCN have higher school absenteeism, which could–at least partly–explain their poorer school performance and lower educational attainment. SHCN-specific targeted interventions may reduce the adverse effects of SHCN on educational outcomes in children.

## Introduction

The number of children with special health care needs (SHCN) due to a chronic health condition has increased over the past decades [1], and the prevalence of SHCN is currently between 12% and 17% among children and adolescents [2, 3]. SHCN due to a chronic health condition are associated with poorer school performance [4], which in turn may adversely impact later educational outcomes, socio-economic status, and general health in adulthood [5].

**Fig 1:**
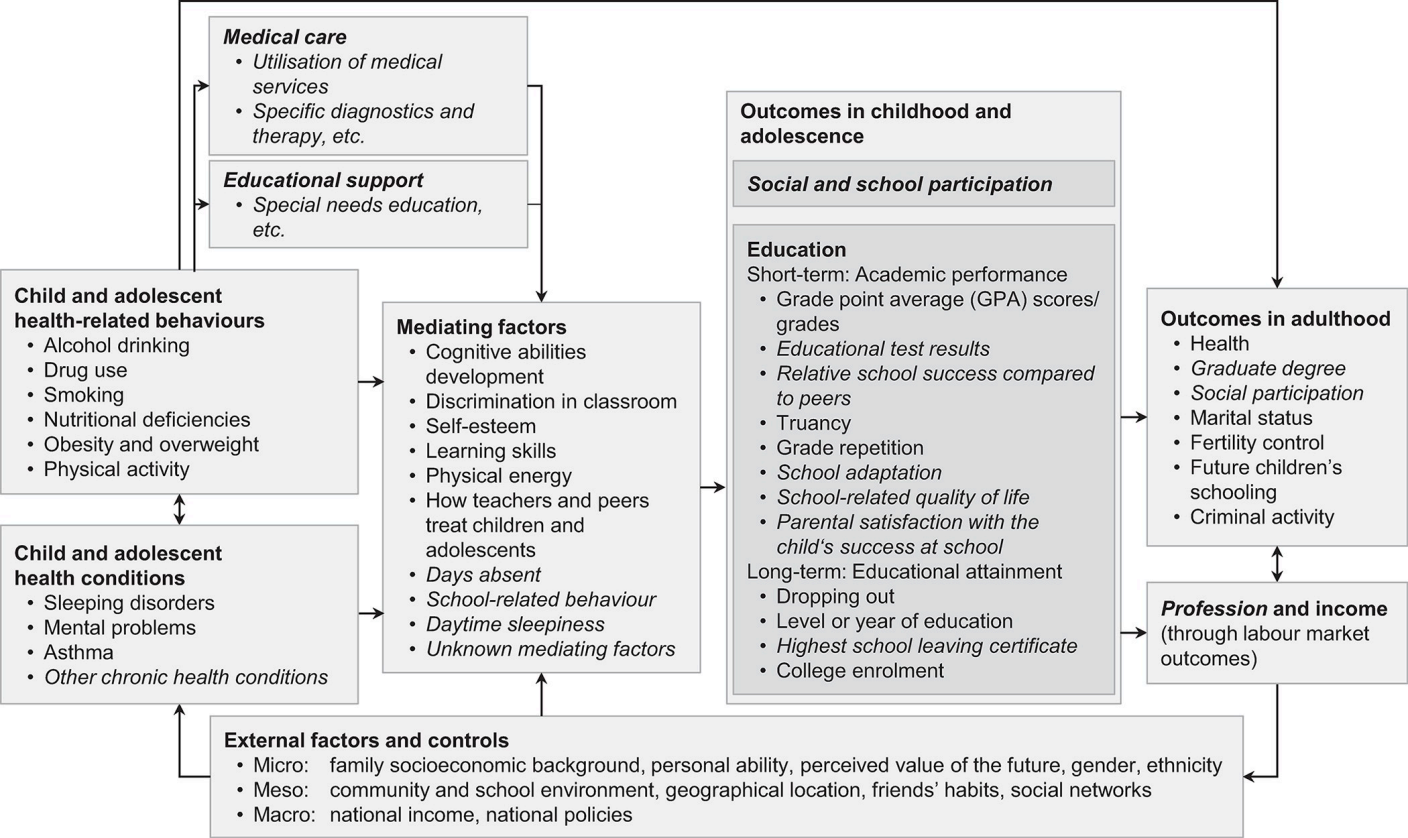
Analytical framework of the association between health and education. Based on Suhrcke and de Paz Nieves [5] with extensions by Dadaczynski [6] and Urschitz et al. [7] indicated in italics.

The relationships between child health and educational outcomes and the potential mediating and moderating factors have been conceptualized in a theoretical framework by Suhrcke and de Paz Nieves [5]. This framework was later modified by Dadaczynski [6] and further extended by our group (Fig 1) by including additional potential mediators, moderators, and further school-related outcomes [7].

Within this framework, school absenteeism plays a crucial role for several reasons: i) It may hamper a child’s ability to build relationships with classmates and keep it from participating in school activities that may stimulate their learning and development, ii) it is an important predictor for academic achievement [8], iii) it has been suggested as a causal link (i.e., mediator) between SHCN and impaired educational outcomes [7], and iv) it may be a potentially modifiable target for specific health-related interventions [9].

There is substantial research showing that school absenteeism is negatively associated with students’ academic achievement in children without SHCN. For instance, Klein et al. [8] studied the predictive power of overall and different types of school absence on academic achievement at the end of compulsory schooling in Scotland (n = 4,419). They found that sickness absence (standardized mean difference (SMD) -0.04) and truancy (SMD -0.04) and to a lesser extent family holidays (SMD -0.03) and exceptional domestic circumstances (SMD -0.02) were associated with academic achievement.

Most of the evidence, however, came from studies in North America, where children with mild to moderate SHCN usually attend regular schools without having the opportunity for supportive school health services as provided in special schools. For European countries such as Germany, where children with physical or mental disabilities, medical complexity and/or severe SHCN are commonly attending special schools with supportive school health services provided by trained nurses and other health professionals, the evidence is scarce. Due to this selection process prior to school entry, it is unclear, if children with SHCN who attend regular schools are still more likely to have poorer educational outcomes than their peers, and, therefore, may require support. In addition, most studies on the association between SHCN and educational outcomes used cross-sectional instead of longitudinal study designs [6], which may have introduced bias. These research gaps motivated the launch of the ikidS project in 2013 (“ich komme in die Schule” (German); I will start school) [7], which aimed to investigate the outcomes of children with SHCN in a regular school setting. We previously showed that a child’s actual need for special health care due to a physical or mental chronic health condition (mean difference -0.95; 95% CI -1.55, -0.35)–and not the sole presence of a physician diagnosis of a condition (0.02; 95% CI -0.38, 0.42)–was associated with poor school performance at the end of first grade [10]. To further elucidate the causal structure of this association, we investigated in the present study the association between SHCN and health-related days absent from school as a marker of a specific form of school absenteeism.

## Materials and methods

### The ikidS project

The present study used data from the population-based prospective cohort study "ikidS". Details on the ikidS cohort have been published elsewhere [7, 10]. In short, first graders within the city of Mainz and the surrounding district of Mainz-Bingen (Rhineland-Palatinate, Germany) that started school in 2015 were enrolled within their pre-school health examination. Parents or guardians of 2,003 out of 3,683 eligible children agreed to participate. To date, data have been collected at six time points beginning at the pre-school health examination prior to school entry. The public health authority of the Mainz-Bingen district provided pre-school health examination data in person-identifying form for participants and in anonymized form for the entire population.

Approval was granted by the ethics committee of the regional medical association of Rhineland-Palatinate (Ethik-Kommission der Landesärztekammer Rheinland-Pfalz, reference number: 837.544.13 (9229-F)), the regional supervisory school authority, and the state representative for data protection in Rhineland-Palatinate. Informed written consent was obtained from legal guardians of all children included in the study.

### Study sample

Children who were eventually enrolled in an elementary school in 2015 were included in the present study. Children with mental disabilities or a recommendation for a special needs school were excluded.

### Assessment of SHCN

Children with SHCN were identified prior to school entry and at the end of first grade according to their needs and use of special health care or functional limitations by a German version of the Children with Special Health Care Needs screener filled out by parents [11]. The screener consists of 14 items assessing five consequences of physical or mental chronic health conditions: (1) use or need of prescription medication, (2) more use or need of medical care, mental health, or educational services than is usual for most children of the same age, (3) functional limitations (i.e., the child being limited or prevented in any way in his or her ability to do the things most children of the same age can do), (4) use or need of specialized therapies (i.e., physical, occupational, or speech therapy), and (5) treatment or counseling for emotional, behavioral, or developmental problems. A SHCN consequence is considered present if i) the child experiences the respective consequence, ii) the consequence is due to a chronic health condition, and iii) the chronic health condition has lasted or is expected to last for at least 12 months. A SHCN due to a chronic health condition is present if at least one of five consequences is confirmed either prior to school entry or at the end of first grade. We further computed the number of SHCN consequences that a child experiences and categorized it into three levels (0, 1, or >1). Physician diagnoses were based on parental report only.

### Assessment of outcome

The outcome was defined as total days absent from school due to illness during first grade and was reported by the child’s classroom teacher at the end of first grade.

### Confounding variables

A directed acyclic graph (DAG) for the association between chronic health condition and days absent from school was constructed to identify a minimally sufficient adjustment set [12]. Potentially confounding variables included gender, immigration status [13, 14], socio-economic status [15], multiple at birth, breastfeeding, chronic health condition in the family, completion of recommended well-child visits, and school location. Definitions and operationalizations of these variables are given elsewhere [10]. The DAG is provided in the S1 Fig.

Besides the overall effect, we studied the independent associations of each of the five SHCN consequences on days absent. Therefore, a second DAG was constructed including the five SHCN consequences as independent variables and days absent as the dependent variable (S2 Fig). This allowed us to identify minimally sufficient adjustment sets for each of the five SHCN consequences. The effect of each SHCN consequence was then adjusted for the above mentioned confounding variables and the respective confounding SHCN consequences listed in Table 1.

**Table 1:** Minimally sufficient adjustment sets to study the independent effect of each SHCN consequence on days absent from school.

Consequence	Minimally sufficient adjustment set*
1. Use or need of prescription medication	Consequence 2 and 5
2. Above average use or need of medical, mental health, or educational services	Consequence 3, 4, and 5
3. Functional limitations compared with others of the same age	No adjustment was necessary
4. Use or need of specialized therapies	Consequence 3 and 5
5. Treatment or counseling for emotional, behavioral, or developmental problems	Consequence 3

*All models were further adjusted for gender, immigration status, socio-economic status, multiple at birth, breastfeeding, chronic health condition in the family, health care utilization, and school location.

### Statistical analysis

Descriptive statistics were based on complete cases only. Distributions of continuous variables are presented as mean and standard deviation (SD) or median and interquartile range (IQR) as appropriate; results of categorical variables are reported as absolute and relative frequencies. Pairwise correlations between the five binary SHCN consequences were given as phi correlation coefficients.

We estimated the effect of SHCN on days absent from school due to illness from a negative binomial regression model (R package *MASS* [16]) adjusted for confounding variables as described above. For the primary analysis, we used a single overall binary indicator for SHCN as the independent variable. For the secondary analysis, we used binary indicators for the five individual SHCN consequences and the 3-level sum of SHCN consequences, respectively, as independent variables. For each effect estimate, the SMD was computed by dividing the difference in predicted days absent between children with and without SHCN by the predicted standard deviation in children without SHCN. Effects are presented as adjusted rate ratios (RR) with 95% confidence intervals (CI) and SMD between children with SHCN compared to children without SHCN (i.e., not experiencing any SHCN consequence).

Missing values for SHCN status, confounding variables, and days absent from school due to illness during first grade were imputed 10 times using multivariate imputation by chained equations and 100 iterations (R package *mice* [17]) which is an adequate method for dealing with values missing at random in longitudinal studies [18]. Only pooled results are reported [19]. All analyses were carried out with R version 4.2.2 [20].

## Results

Of 2,003 ikidS participants, 1,921 were included in the present analysis (96% of the cohort, 52% of the study population); reasons for exclusion were deferral from school entry (*n* = 51), recommendation for a special needs school (*n* = 27), and mental disability (*n* = 4) (S3 Fig). Children included in the present analysis had similar characteristics compared to the study population except that they were less likely to come from immigrant families (S1 Table).

### Children with SHCN

In total, 202 children (14% of the analysis sample) had SHCN either prior to school entry or at the end of first grade; of these, 43% had SHCN at both time points. Seventy-nine percent of children with SHCN had at least one medical diagnosis that might negatively affect school performance. The most frequent diagnoses were asthma (22%), attention-deficit hyperactivity disorder (ADHD; 17%), and atopic dermatitis (16%) (S2 Table). Compared to the study population, children with SHCN were more likely to be male, come from non-immigrant families, be formula-fed, have mothers with and fathers without high school diploma, and attend school in the rural district of Mainz-Bingen (Table 2).

**Table 2:** Characteristics of children included in the study sample and children with special health care needs*.

	Study sample	Children with special health care needs
	(*n* = 1,921)	(*n* = 202)
**Child**		
Gender		
Male	988 (51.4)	118 (58.4)
Female	933 (48.6)	84 (41.6)
Missing, *n*	0	0
Age at preschool health examination (y), mean (SD)	5.9 (0.4)	5.9 (0.4)
Missing, *n*	0	0
Immigration status		
Yes	428 (23.5)	19 (9.9)
No	1396 (76.5)	172 (90.1)
Missing, *n*	97	11
Multiple at birth		
Yes	57 (3.0)	7 (3.6)
No	1842 (97.0)	189 (96.4)
Missing, *n*	22	6
Breastfeeding		
Not at all	304 (16.5)	42 (22.0)
Up to 6 months	741 (40.2)	70 (36.6)
More than 6 months	796 (43.2)	79 (41.4)
Missing, *n*	80	11
**Family**		
Abitur (A level exams)† Mother		
Yes	1095 (61.9)	123 (65.1)
No	673 (38.1)	66 (34.9)
Missing, *n*	153	13
Abitur (A level exams)† Father		
Yes	1017 (60.0)	102 (55.7)
No	677 (40.0)	81 (44.3)
Missing, *n*	227	19
**School location**		
District of Mainz-Bingen (rural)	996 (51.8)	125 (61.9)
City of Mainz	925 (48.2)	77 (38.1)
**Days absent from school due to illness during first grade**		
0–4 days	733 (53.8)	75 (48.7)
5–9 days	403 (29.6)	44 (28.6)
10–14 days	143 (10.5)	22 (14.3)
≥15 days	84 (6.2)	13 (8.4)
Missing, *n*	558	48

* Unless otherwise stated, values are expressed as n (%). % relate to non-missing values. Abbreviations: SD, standard deviation.

† Including advanced technical college entrance qualification.

The most frequent SHCN consequences were above average use or need of medical, mental health, or educational services and use or need of prescription medication (each 46% among children with SHCN), treatment or counseling for emotional, behavioral, or developmental problems (41%), use or need of specialized therapies (40%), and functional limitations compared with others of the same age (19%). Pairwise correlations (phi coefficient) were highest between above average use or need of medical, mental health, or educational services and each of the other consequences (range: 0.45; 0.53) and between use or need of specialized therapies and treatment or counseling for emotional, behavioral, or developmental problems (0.51) (Table 3).

**Table 3:** Pairwise correlations between consequences of chronic health conditions assessed by the children with special health care needs screener*.

Consequence	1	2	3	4	5
1 Use or need of prescription medication	1.00				
2 Above average use or need of medical, mental health, or educational services	0.45	1.00			
3 Functional limitations compared with others of the same age	0.22	0.52	1.00		
4 Use or need of specialized therapies	0.20	0.49	0.35	1.00	
5 Treatment or counseling for emotional, behavioral, or developmental problems	0.23	0.53	0.33	0.51	1.00

* Pairwise phi correlation coefficients are shown.

About half of the children with SHCN experienced only one consequence (52% of children with SHCN), while the other half experienced more than one (2: 21%, 3: 12%, 4: 10%, 5: 4%).

### Overall effect of SHCN on days absent from school due to illness

**Fig 2:**
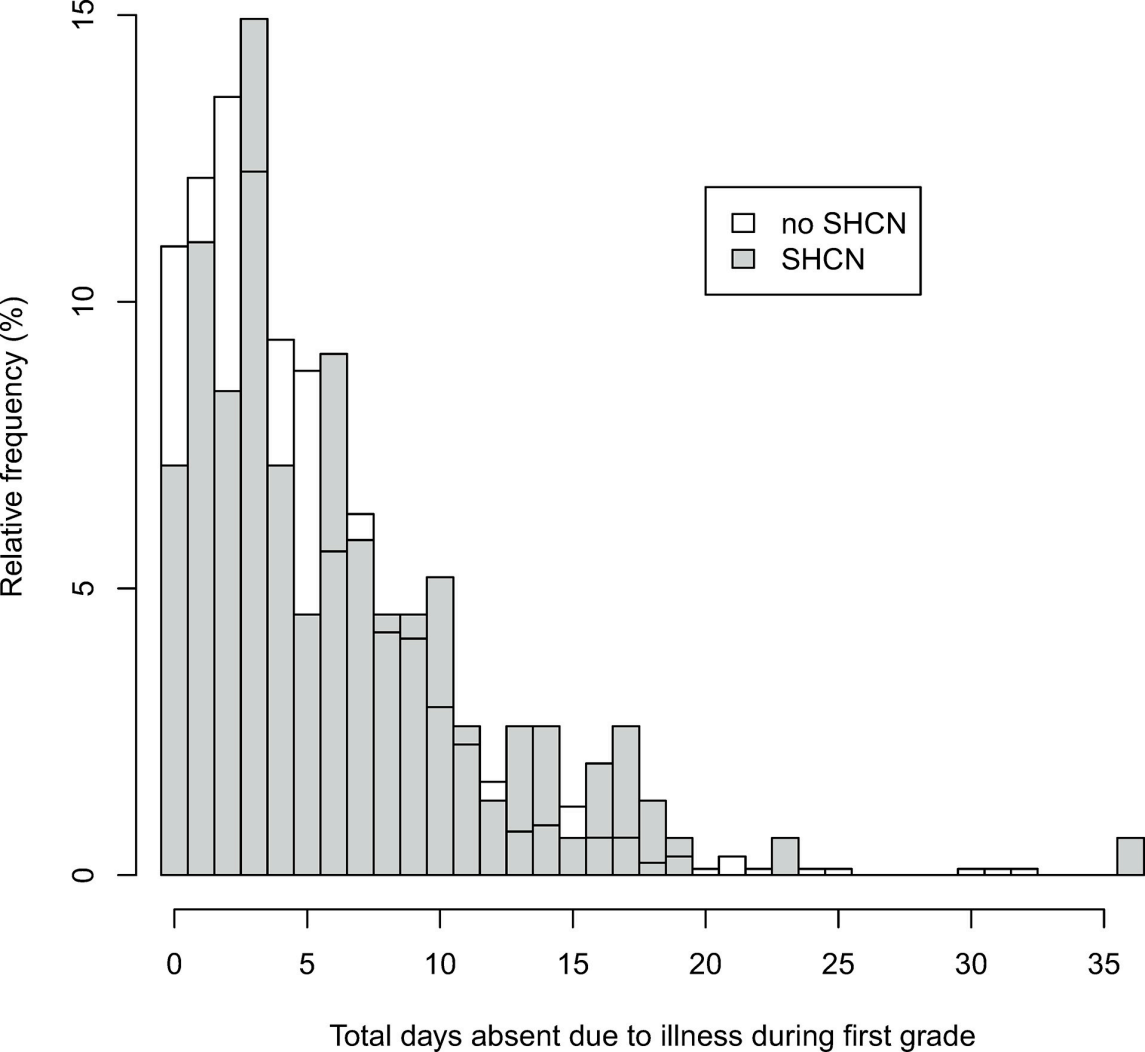
Distribution of days absent from school due to illness during first grade between children with and children without special health care needs.

Children with SHCN had slightly more days absent from school than children without SHCN (median (IQR) 5 (7) vs. 4 (5)). The distribution of days absent for these children was slightly shifted towards more days absent from school (Table 2, Fig 2). In the adjusted analysis, the rate of days absent from school was 1.37 times higher (95% CI 1.16, 1.62; SMD 0.41) for children with SHCN compared to children without SHCN (Table 4).

**Table 4:** Associations between special health care needs and days absent from school due to illness during first grade (n = 1,921)*.

		Rate Ratio, adjusted†	[95% CI]	SMD
**Overall SHCN**				
No SHCN		(ref)		
SHCN		1.37	[1.16; 1.62]	0.40
**Individual SHCN consequences**				
Use or need of prescription medication	No	(ref)		
	Yes	1.13	[0.89; 1.45]	0.15
Above average use or need of medical, mental health, or educational services	No	(ref)		
	Yes	1.13	[0.88; 1.46]	0.14
Functional limitations compared with others of the same age	No	(ref)		
	Yes	1.58	[1.15; 2.17]	0.64
Use or need of specialized therapies	No	(ref)		
	Yes	1.17	[0.88; 1.55]	0.19
Treatment or counseling for emotional, behavioral, or developmental problems	No	(ref)		
	Yes	1.38	[1.14; 1.68]	0.41
**Number of SHCN consequences experienced**				
0 consequences		(ref)		
1 SHCN consequence		1.29	[1.08; 1.54]	0.32
>1 SHCN consequences		1.48	[1.23; 1.77]	0.52

* Abbreviations: CI, confidence interval; SHCN, special health care needs; SMD, standardized mean difference.

† Adjusted for gender, immigration status, socio-economic status, multiple at birth, breastfeeding, chronic health condition in the family, health care utilization, and school location.

### Effect of individual SHCN consequences and number of consequences experienced on days absent from school due to illness

The strongest association between individual SHCN consequences and days absent from school due to illness was found for functional limitations compared with others of the same age (RR 1.58; 95% CI 1.15, 2.17; SMD 0.64) and treatment or counseling for emotional, behavioral, or developmental problems (RR 1.38; 95% CI 1.14, 1.68; SMD 0.41; Table 4). The overall effect of SHCN on days absent from school due to illness was primarily driven by children who experienced more than one consequence (RR 1.48; 95% CI 1.23, 1.77; SMD 0.52) and less by children who experienced only one consequence (RR 1.29; 95% CI 1.08, 1.54; SMD 0.32; Table 4).

## Discussion

The results of this prospective cohort study suggest that children with SHCN miss more school days compared to children without SHCN, which is largely in line with evidence from other countries. In their systematic meta-review, Lum et al. [4] found that children with six defined chronic health conditions have greater school absence; 82 out of 88 studies reported an association with absence. By contrast to other studies that used a diagnosis-based framework for chronic health conditions, our study identified children on the basis of their actual needs or use of special health care services or functional limitations. This consequence-based framework covers varying diagnoses and disease severities as well as children without a definite medical diagnosis [21]. In our sample of children with SHCN, the most frequent diagnoses were asthma, ADHD, and atopic dermatitis, which may be–at least partly–responsible for the observed association between SHCN and school absence.

We also found that children with functional limitations, treatment or counseling for emotional, behavioral, or developmental problems, or those who experienced more than one SHCN consequence were at highest risk for school absenteeism. In keeping with the latter finding, Lum et al. [4] also reported that children with more severe diseases or frequent hospitalizations were at higher risk for school absence. The Children with Special Health Care Needs screener does not inherently measure the severity of disease. However, being limited or prevented in the ability to do the same tasks most children of the same age can do (i.e., having functional limitations) and experience two or more SHCN consequences can be seen as an indicator of a more severe disease or medical complexity.

Our results support the hypothesis that school absenteeism could be an important causal link in the relationship between SHCN and poor school performance in early childhood. In a Scottish study, Klein et al. examined associations between different forms of absenteeism and achievement. They found that all forms of school absences (i.e. truancy, sickness absence, exceptional domestic circumstances, and family holidays) were negatively associated with achievement at the end of compulsory and postcompulsory schooling [8]. The magnitude of the effect for truancy was similar to the effect for sickness absence, which supports the importance of school absenteeism due to health-related problems as investigated in the present study.

School absenteeism may therefore be a promising target for health-related interventions in children with SHCN. Generic interventions with specific measures for individual conditions aiming to reduce school absenteeism may potentially mitigate the negative effects of SHCN on educational outcomes. For example, a nurse-delivered asthma program to elementary schools led to a significant reduction in school absence and interrupted activities in children with asthma [9]. The effects of the program on school performance, however, were not reported. Future studies on health-related interventions for children with SHCN should examine their effects on both school absenteeism *and* school performance.

### Strengths and limitations

Most studies on the association between health and educational outcomes have been conducted in the United States [4, 22, 23]. This is the first population-based, longitudinal study from Germany and one of the first in continental Europe. The study sample was largely representative of the underlying study population and covered rural and urban areas, which makes our findings generalizable to regular schools in other areas in Germany with a similar population structure.

For the identification of affected children, we used a comprehensive consequences-based concept of chronic health conditions, the actual need for special health care as measured by the Children with Special Health Care Needs screener. This approach defines the presence of a relevant chronic condition through the parent-reported actual need or use of these services and perceived functional impairments. Thus, children with chronic health conditions but *without* current SHCN were not identified and remained in the comparison group for analysis. Any remaining misclassification may not be important, since a previous study from the ikidS project suggested that a chronic health condition *without* actual SHCN is not related to poorer school performance [10].

In many federal states of Germany, most children with severe disabilities and medical complexity attend special schools; our sample of children attending regular public schools, therefore, included only children with mild to moderate physical, developmental, and mental health conditions, as well as children who are currently medically evaluated and have no definitive diagnosis. Thus, our findings can not be generalized to children with SHCN attending special schools.

In our sample of children with SHCN, the majority of children had asthma or ADHD. Due to this heterogeneity, the effect on days absent from school reported in this study should be interpreted as the pooled effect of individual effects of the underlying conditions.

### Conclusions

In a sample from children attending regular schools without children with severe disabilities and medical complexity, children with mild to moderate SHCN may still have more school days absent compared to children without SHCN. This is of importance as regular schools–in contrast to special schools–are usually not equipped with school health services and affected children are not identified and supported.

Our findings indicate issues with the allocation of children with SHCN to regular schools and the uptake and the effectiveness of existing medical care as well as educational support concepts for children with SHCN in regular schools. Our results also suggest that days absent should be monitored at school in particular in children with SHCN and reasons for the absence should be addressed.

Existing school health services may be extended with measures tailored for individual health conditions with the goal to reduce school absenteeism. In countries, where school health services are not yet fully established (as in Germany), our results may be used to indicate the urgent need for such services, especially for children with SHCN.

Finally, the fast growing body of evidence on school health interventions for children with SHCN should be evaluated regularly, and effective interventions should be identified, established in policies, and transferred to school health practice. Scoping reviews, implementation guidelines, and best practices would be in particular helpful to achieve those goals.

## Supporting information

S1 Fig Directed acyclic graph of the effect of special health care needs on days absent from school. Variables in red indicate the minimally sufficient adjustment set. Paths in grey were blocked by the adjustment set. Abbreviations: Accid, accidents; AgeSchEntr, age at school entry; BMI, body mass index; Breastf, breastfeeding; CHCfam, chronic health condition in the family; DaysAbs, days absent from school; EducOrient, educational orientation; HealCarUtiliz, health care utilization; HospStay, hospital stay; Infect, infections; MH, mental health; MigrBackgr, migrant background; Multip, multiple at birth; Nutr, nutrition; PhysAct, physical activity; PreAcadSkills, pre-academic skills; SES, socio-economic status; SHCN, special health care needs; Sibl, siblings; SinglParFam, single-parent family; SocInt, social integration; and WorkTimMod, work time model.
(TIF)

S2 Fig Directed acyclic graph of the effect of single special health care needs consequences on days absent from school. Abbreviations: SHCN, special health care needs, and DAS, days absent from school.
(TIF)

S3 Fig Flow chart of ikidS participants. (TIF)

S1 Table Characteristics of children within the study region (population), children enrolled into the study (participants), and children included in the study sample. (DOCX)

S2 Table Diagnoses among children with special health care needs (*n* = 202). (DOCX)
