# Supplementary figures and images for: School absenteeism in children with special health care needs. Results from the prospective cohort study ikidS
Source: PLoS One. 2023 Jun 23;18(6):e0287408. doi: 10.1371/journal.pone.0287408 (PMC10289337; doi:10.1371/journal.pone.0287408)

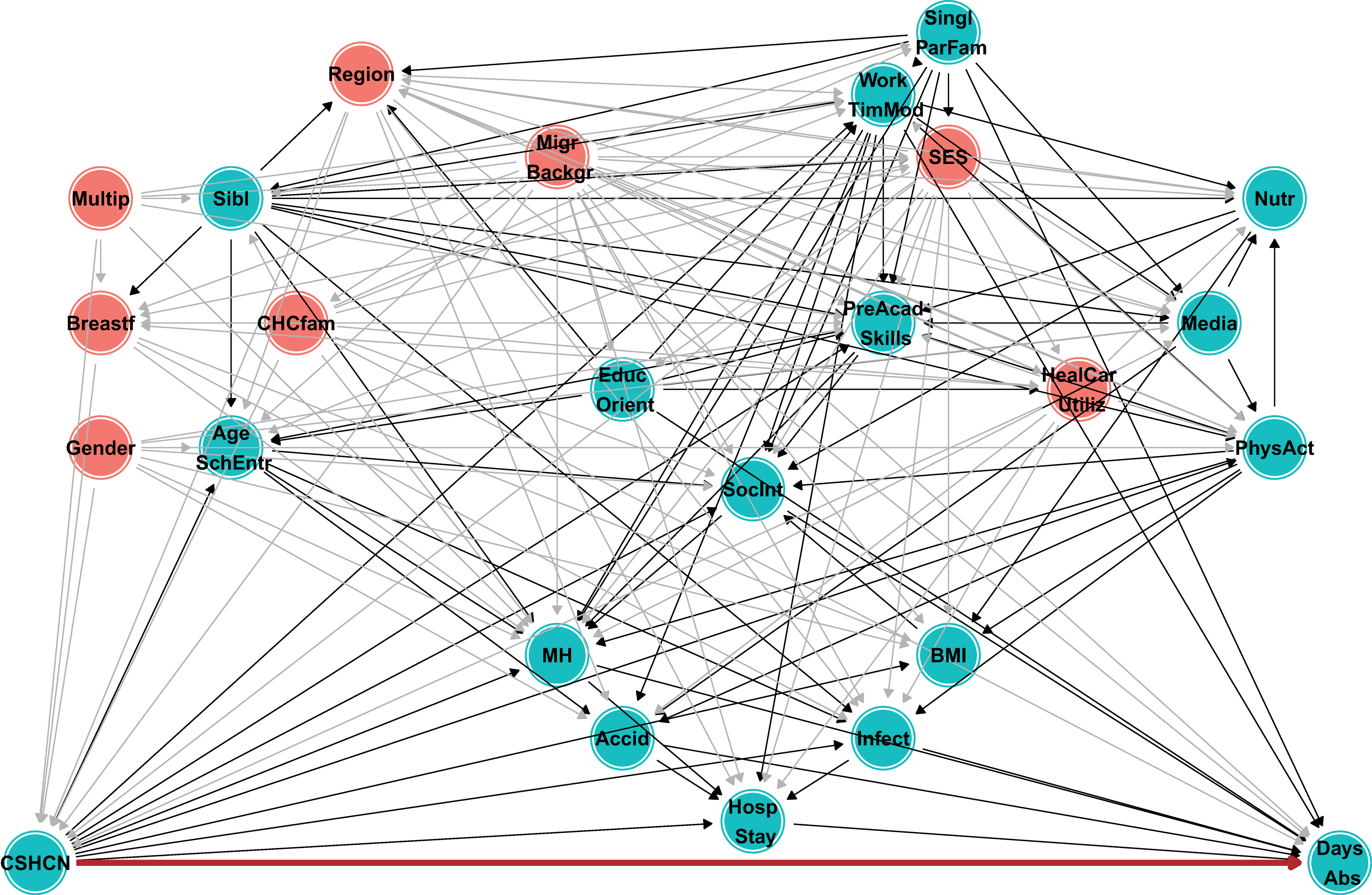

Supplement: S1 Fig — Variables in red indicate the minimally sufficient adjustment set. Paths in grey were blocked by the adjustment set. Abbreviations: Accid, accidents; AgeSchEntr, age at school entry; BMI, body mass index; Breastf, breastfeeding; CHCfam, chronic health condition in the family; DaysAbs, days absent from school; EducOrient, educational orientation; HealCarUtiliz, health care utilization; HospStay, hospital stay; Infect, infections; MH, mental health; MigrBackgr, migrant background; Multip, multiple at birth; Nutr, nutrition; PhysAct, physical activity; PreAcadSkills, pre-academic skills; SES, socio-economic status; SHCN, special health care needs; Sibl, siblings; SinglParFam, single-parent family; SocInt, social integration; and WorkTimMod, work time model. (TIF) [file pone.0287408.s001.tif]

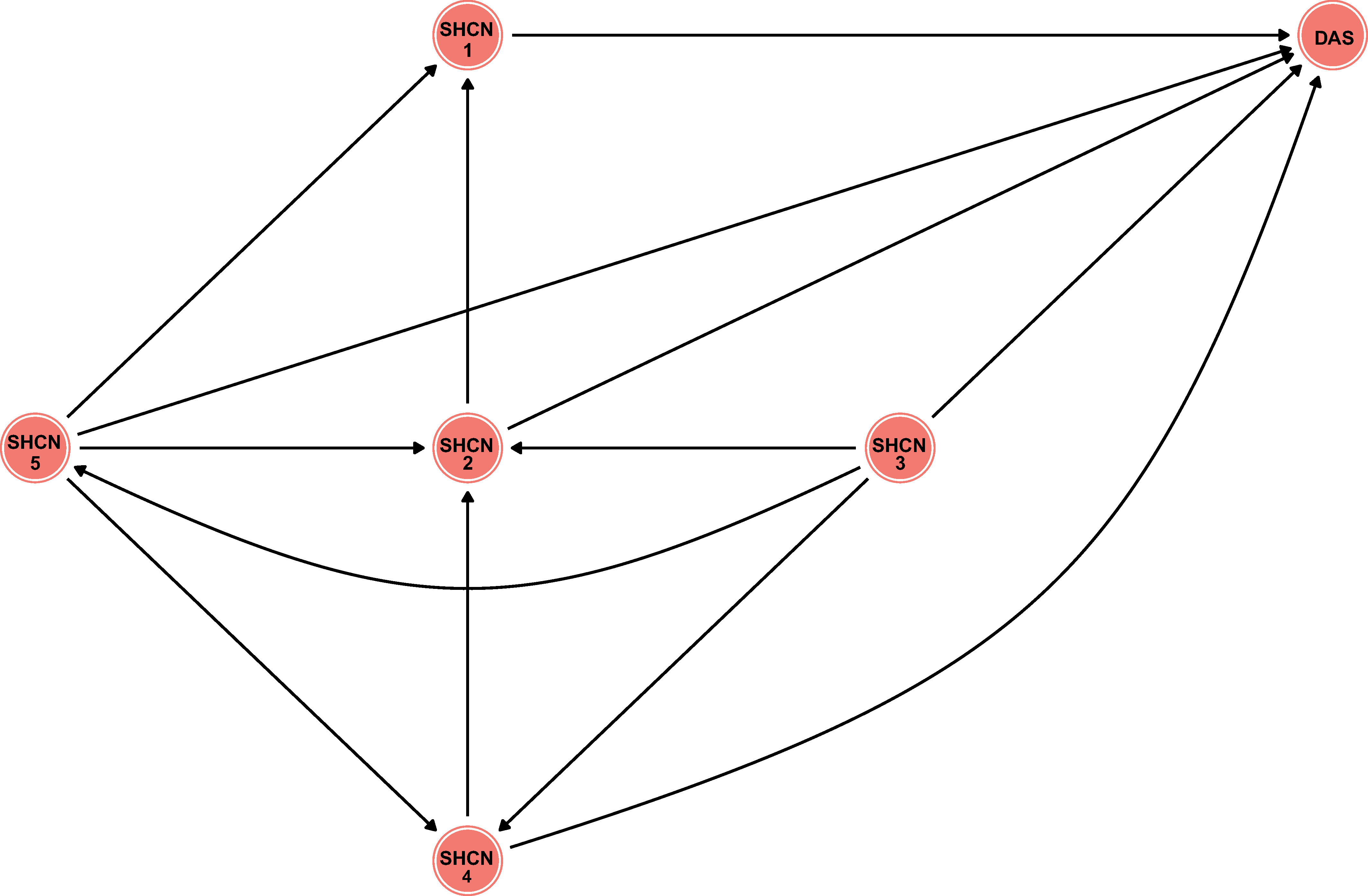

Supplement: S2 Fig — Abbreviations: SHCN, special health care needs, and DAS, days absent from school. (TIF) [file pone.0287408.s002.tif]

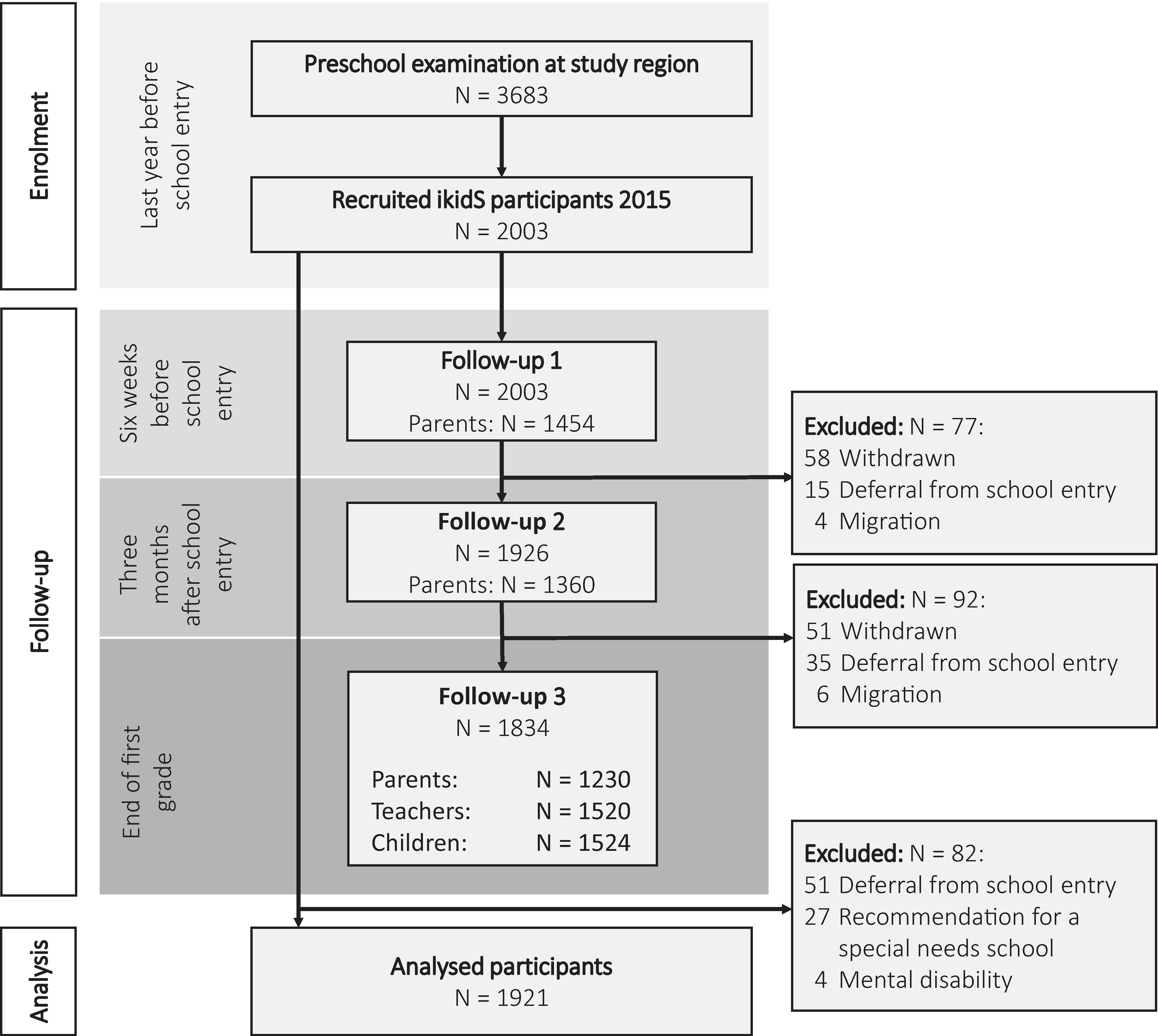

Supplement: S3 Fig — (TIF) [file pone.0287408.s003.tif]
