# Supplementary material for: School absenteeism in children with special health care needs. Results from the prospective cohort study ikidS
Source: PLoS One. 2023 Jun 23;18(6):e0287408. doi: 10.1371/journal.pone.0287408 (PMC10289337; doi:10.1371/journal.pone.0287408)
Supplement: S1 Table — (DOCX) [file pone.0287408.s004.docx]

| **S1 Table. Characteristics of children within the study region (population), children enrolled into the study (participants), and children included in the study sample*** | | | |
| --- | --- | --- | --- |
|  | **Study population** | **Study participants** | **Study sample** |
|  | (n = 3,683) | (n = 2,003) | (n = 1,921) |
| **Child** |  |  |  |
| Gender |  |  |  |
| Male | 1,909 (51.9) | 1,042 (52.0) | 988 (51.4) |
| Female | 1,767 (48.1) | 961 (48.0) | 933 (48.6) |
| Missing, n | 7 | 0 | 0 |
| Age at preschool health examination (y), mean (SD) | 5.9 (0.4) | 5.9 (0.4) | 5.9 (0.4) |
| Missing, n | 1 | 0 | 0 |
| Immigration status |  |  |  |
| Yes | 822 (25.5) | 452 (23.8) | 428 (23.5) |
| No | 2,397 (74.5) | 1,449 (76.2) | 1396 (76.5) |
| Missing, n | 464 | 102 | 97 |
| Multiple at birth |  |  |  |
| Yes | 105 (2.9) | 63 (3.2) | 57 (3.0) |
| No | 3,542 (97.1) | 1,916 (96.8) | 1842 (97.0) |
| Missing, n | 36 | 24 | 22 |
| Breastfeeding |  |  |  |
| Not at all | 573 (17.5) | 326 (17.0) | 304 (16.5) |
| Up to 6 months | 1,312 (40.2) | 776 (40.4) | 741 (40.2) |
| More than 6 months | 1,382 (42.3) | 817 (42.6) | 796 (43.2) |
| Missing, n | 416 | 84 | 80 |
| **Family** |  |  |  |
| Abitur (A level exams)† Mother |  |  |  |
| Yes | 1,825 (60.4) | 1,117 (60.7) | 1095 (61.9) |
| No | 1,196 (39.6) | 724 (39.3) | 673 (38.1) |
| Missing, n | 662 | 162 | 153 |
| Abitur (A level exams)† Father |  |  |  |
| Yes | 1,768 (61.0) | 1,042 (59.1) | 1017 (60.0) |
| No | 1,131 (39.0) | 721 (40.9) | 677 (40.0) |
| Missing, n | 784 | 240 | 227 |
| **School location** |  |  |  |
| District of Mainz-Bingen (rural) | 1,910 (51.9) | 1,044 (52.1) | 996 (51.8) |
| City of Mainz | 1,773 (48.1) | 959 (47.9) | 925 (48.2) |
| * Unless otherwise stated, values are expressed as n (%). % relate to non-missing values. SD indicates standard deviation.  † Including advanced technical college entrance qualification. | | | |
