# Supplementary material for: School absenteeism in children with special health care needs. Results from the prospective cohort study ikidS
Source: PLoS One. 2023 Jun 23;18(6):e0287408. doi: 10.1371/journal.pone.0287408 (PMC10289337; doi:10.1371/journal.pone.0287408)
Supplement: S2 Table — (DOCX) [file pone.0287408.s005.docx]

| **S2 Table. Diagnoses among children with special health care needs (*n* = 202) *** | |
| --- | --- |
| Diagnosis |  |
| Any diagnosis | 159 (78.7) |
| No diagnosis | 43 (21.3) |
|  |  |
| Asthma | 45 (22.3) |
| Attention-deficit hyperactivity disorder (ADHD) | 35 (17.3) |
| Atopic dermatitis | 33 (16.3) |
| Premature birth | 30 (14.9) |
| Sleepiness | 29 (14.4) |
| Hay fever | 27 (13.4) |
| Underweight | 27 (13.4) |
| Overweight | 16 (7.9) |
| Language development disorder | 14 (6.9) |
| Emotional problems | 12 (5.9) |
| * Values are expressed as n (%). % relate to non-missing values. The table is limited to the ten most frequent diagnoses among children with special health care needs. One child may have several diagnoses. | |
